# Renal Dysfunction Across Etiologies of Chronic Liver Disease: A Cross-Sectional Study From a Tertiary Care Center

**DOI:** 10.7759/cureus.110866

**Published:** 2026-06-15

**Authors:** Asha Saranya Rapeti, Rakesh Pilla, Gayathri Chouthai, Abhinav Maharaj Thota, Mrinal Shashidhar, Chaithanya Shanthi Vanamada

**Affiliations:** 1 Internal Medicine, Medicover Hospital, Visakhapatnam, IND

**Keywords:** cld, gfr, masld, renal dysfunction, subclinical kidney disease

## Abstract

Background: Chronic liver disease (CLD) is a rapidly growing public health challenge globally. Kidney complications are a frequent and serious consequence of CLD, significantly increasing the risk of illness and death. Despite this, renal involvement in newly diagnosed or previously untreated CLD remains incompletely understood.

Objective: This study evaluated baseline renal function in patients with newly diagnosed CLD and examined the association between specific etiologies, particularly metabolic dysfunction-associated steatotic liver disease (MASLD), and patterns of overt or subclinical renal dysfunction.

Methods: A cross-sectional observational study was conducted involving 79 adults newly diagnosed with untreated CLD at a tertiary care hospital in India between March 2023 and August 2024. Individuals with known chronic kidney disease, prior treatment for decompensated CLD, or recent exposure to nephrotoxic medications were excluded. Renal function assessment included standard blood tests, 24-hour urine collections, and estimated glomerular filtration rate (GFR) calculations. To evaluate suspected subclinical renal injury, renal biopsy was performed in 40 clinically selected patients with proteinuria, active urinary sediment, reduced GFR, or suspected glomerular disease.

Results: The cohort was predominantly composed of middle-aged men, with a mean age of 49.26 years. Alcohol-related liver disease was the most common etiology, affecting 39 patients (49.4%), followed by MASLD (14 patients, 17.7%) and viral hepatitis (B and C combined) (10 patients, 12.7%). Despite 71 patients (88.6%) exhibiting normal serum creatinine levels, biopsies (n=40) revealed substantial underlying structural abnormalities. Non-glomerular lesions were identified in 35 (87.5%) of biopsied cases, while IgA nephropathy was the most frequent glomerular pathology, present in 18 (45.0%) biopsied patients. Overall, nine (11.4%) of the cohort had a reduced GFR, defined as <60 mL/min/1.73m². Patients with MASLD demonstrated a significantly higher prevalence of reduced GFR compared to those with alcohol-related disease, i.e., four (28.6%) versus two (5.1%), corresponding to more than a sevenfold increase in the odds of renal dysfunction.

Conclusion: Subclinical structural renal damage may be common in the early stages of CLD and often precedes detectable changes in routine blood tests. MASLD is associated with a markedly increased risk of reduced GFR compared to other etiologies. Early and concurrent assessment of renal function in patients with newly diagnosed CLD is critical for identifying subclinical impairment and informing timely clinical management.

## Introduction

Chronic liver disease (CLD), which includes metabolic dysfunction-associated steatotic liver disease (MASLD), alcohol-related liver disease (ALD), and chronic viral hepatitis, is an increasing public health concern worldwide. The impact is especially significant in developing regions like India, where projections indicate that a large proportion of adults could be affected by these related conditions by 2040 [1]. The rising rates of MASLD are particularly important given its widespread complications [2].

Renal complications are frequent and serious in CLD, contributing to increased morbidity and mortality. The interplay between hepatic and renal systems is multifaceted. Progressive hepatic dysfunction leads to alterations in hemodynamics, inflammation, and metabolic processes that adversely affect renal function. Both functional and structural renal injuries may occur. In addition to oxidative stress and vascular abnormalities, factors such as insulin resistance, dyslipidemia, and renal steatosis are increasingly recognized as significant contributors to kidney injury in this population [3].

Accurate assessment of renal function in individuals with CLD is challenging. Serum creatinine is frequently unreliable due to reduced muscle mass and decreased creatinine production in cirrhotic patients. Consequently, recent studies advocate for the use of more precise methods to estimate glomerular filtration rate (GFR), including timed urine collections and advanced estimation formulas, to better characterize renal status [4]. These complexities underscore the necessity of evaluating hepatic and renal function concurrently, particularly in patients with metabolic risk factors.

While severe liver disease is known to cause obvious kidney problems, the effects of early-stage CLD on the kidneys are not fully understood. Chronic kidney disease (CKD) occurs in 3% to 47% of people with cirrhosis, with higher rates in those with more severe disease and other health issues [5]. Recent research shows that the cause of liver disease affects kidney outcomes. MASLD, in particular, is linked to a higher risk of CKD, likely because of related conditions like diabetes and high blood pressure [6,7]. As a result, patients with MASLD may have faster kidney decline than those with viral or ALD.

To address this knowledge gap, this cross-sectional observational study aims to evaluate the prevalence and etiology-specific patterns of renal dysfunction in patients with CLD at a tertiary care center in Northern Andhra Pradesh, India. By systematically assessing clinical characteristics, biochemical parameters, and GFR, the study aims to clarify the relative contributions of different CLD etiologies, particularly MASLD, to renal impairment. The primary outcome was defined as a reduced estimated GFR (eGFR) <60 mL/min/1.73 m² by CKD-EPI 2021 [8]. Secondary outcomes included proteinuria, abnormal urinary sediment, and renal histopathological findings in the biopsy subgroup.

## Materials and methods

Study design and setting

This cross-sectional study was conducted at the Department of General Medicine at Medicover Multi-Specialty Hospital, Visakhapatnam, India, from March 2023 to August 2024.

Study population 

A total of 267 consecutive patients were admitted with CLD, and the study focused on newly diagnosed, untreated individuals with CLDs caused by alcohol, MASLD, viral hepatitis, or autoimmune and metabolic conditions.

Inclusion and Exclusion Criteria

Patients presenting with first-time decompensating events (e.g., new-onset ascites) were included to allow baseline diagnostic evaluation prior to treatment.

Table 1 shows the excluded cohort list.

**Table 1:** Summary of participant exclusions and reasons CLD: Chronic Liver Disease; CKD: Chronic Kidney Disease. ¹Includes diuretics and vasoactive drugs.

S.no	Exclusion reason	Number
1	Known decompensated CLD on treatment	144
2	On nephrotoxic medication	15
3	On other medications that can affect renal hemodynamics^1^	19
4	Known CKD	10

Definitions

Clinical outcomes and conditions were classified using standard diagnostic frameworks. CKD and acute kidney injury (AKI) were defined according to the Kidney Disease: Improving Global Outcomes (KDIGO) criteria [9]. Specifically, CKD was defined as an eGFR <60 mL/min/1.73m² persisting for more than three months, or the presence of established markers of kidney damage. AKI was defined by an increase in serum creatinine ≥0.3 mg/dL in 48 h or ≥1.5 times the baseline value within seven days. Additionally, proteinuria was defined as total protein excretion ≥150 mg/day, while significant proteinuria was specifically designated as an excretion level ≥500 mg/day.

Study Cohort

**Figure 1:**
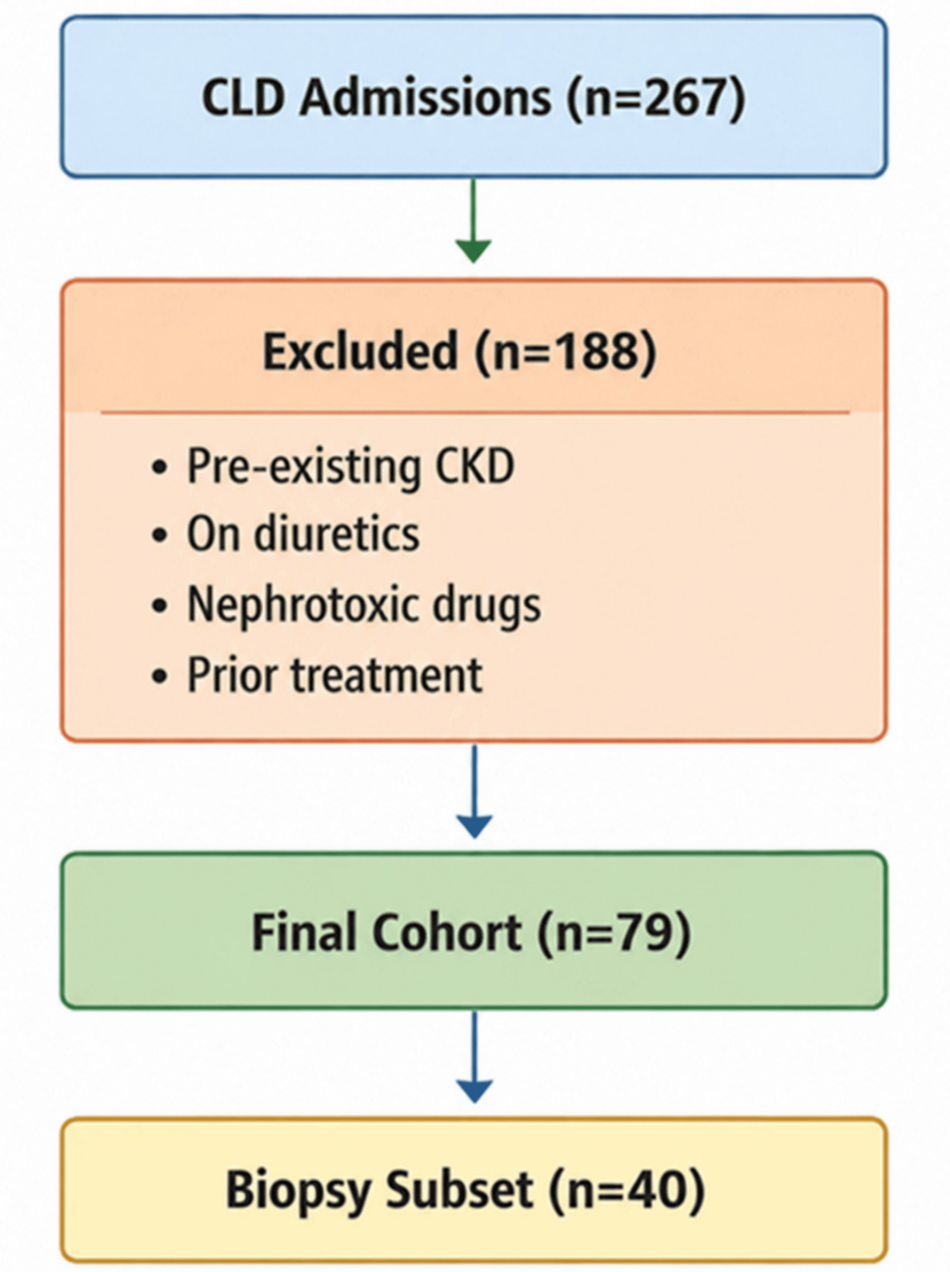
Flow chart representing the cohort selection CLD: Chronic Liver Disease; CKD: Chronic Kidney Disease.

After applying the predefined exclusion criteria, 79 consecutive eligible patients remained in the final study group, as detailed in Figure 1. No additional selection processes or demographic restrictions were applied beyond the stated criteria.

Renal biopsy subset

Percutaneous renal biopsy was performed in 40 patients to investigate suspected renal pathology. The indications for performing a biopsy included persistent proteinuria greater than 500 mg/day, an active urinary sediment (≥5 red blood cells or casts per high-power field), a reduced measured GFR despite a normal serum creatinine level, or a strong clinical suspicion of glomerular disease. Strict safety criteria were enforced, and the biopsy was executed only when all of the following parameters were met: a platelet count ≥100,000/mm³, an international normalised ratio (INR) ≤1.4, a hemoglobin level ≥8 g/dL, and a controlled blood pressure below 140/90 mmHg. Furthermore, patients were excluded if they presented with uncontrolled ascites, active infection, sepsis, or had received recent anticoagulant or antiplatelet therapy. For individuals presenting with ascites, a therapeutic paracentesis was performed prior to the biopsy procedure if clinically indicated.

Biopsy Procedure

Biopsies were performed under ultrasound guidance using a 16-gauge automated needle in a monitored clinical setting. No major bleeding events or serious complications were observed.

Ethical considerations

The Institutional Ethics Committee, Medicover Hospital, issued approval 12/IEC/MH-HC/2022/17. Experts from General Medicine and Gastroenterology were consulted. Written consent in the local language was obtained from participants before enrollment. All procedures followed institutional guidelines. Separate informed consent was obtained for renal biopsy.

Data collection and clinical assessment 

At enrollment, participants underwent interviews regarding medical history, alcohol consumption, and acute symptoms, including jaundice, dark urine, hematemesis, or melena.

A comprehensive physical examination was conducted to identify hepatic abnormalities and signs of portal hypertension. The assessment included evaluation for cutaneous manifestations, features of decompensation, endocrine disturbances, and clinical markers of portal hypertension.

Laboratory investigations 

All patients received blood tests and liver function tests according to the study protocol.

Etiology was determined by serological testing, including hepatitis B surface antigen (HBsAg) for viral hepatitis and autoimmune markers (antinuclear antibodies (ANA), liver-kidney microsomal (LKM)-1, LKM-2, LKM-3).

For patients who had not yet received treatment and had developed new ascites, fluid analysis was performed to confirm the cause and assess baseline severity.

Renal function assessment 

Kidney function was assessed using 24-hour urine collections to measure volume and protein excretion. Creatinine clearance was calculated, along with estimates from the Cockcroft-Gault [10] and CKD-EPI Creatinine (2021) [8] formulas. All measured 24-hour creatinine clearance values were mathematically corrected for body surface area and indexed to 1.73 m² prior to comparative analysis with the eGFR formulas.

Imaging and endoscopic evaluation

All participants underwent abdominal ultrasound to evaluate the liver, kidneys, ureters, and bladder. They also had an upper gastrointestinal endoscopy to look for varices and assess how severe portal hypertension was.

Statistical analysis

Data analysis included both descriptive and inferential statistics. Categorical data were shown as frequencies and percentages, while continuous data were given as mean and standard deviation. The association between liver disease and kidney problems was tested using Fisher’s exact test, odds ratios with 95% confidence intervals were calculated comparing the rates of reduced GFR (less than 60 mL/min/1.73m²) across groups, with ALD as the reference. A p-value below 0.05 was considered significant.

## Results

Cohort demographics and hepatic profile

This study evaluated 79 patients with CLD. Fifty-three were men, with an average age of 49.26 years. ALD was the most common cause (39 patients, 49.4%), followed by MASLD (14 patients, 17.7%), chronic hepatitis B (eight patients, 10.1%), autoimmune hepatitis (four patients, 5.1%), and smaller numbers with hepatitis C (two patients, 2.5%), Wilson's disease (one patient, 1.3%), cryptogenic cirrhosis (four patients, 5.1%), and combined/overlapping causes (five patients, 6.3%).

Most patients had moderate increases in bilirubin, with 47 (59.5%) showing serum total bilirubin between 2 and 4 mg/dL. Slightly raised alanine aminotransferase (ALT) levels (100-200 IU/L) were seen in 41 patients (51.9%). Low albumin levels (below 3 g/dL) were found in 66 patients (83.5%). Ultrasound showed a portal vein diameter of 13-15 mm in 58 (73.4%) of cases. For disease severity, 59 (74.7%) were Child-Pugh Class B, 12 (15.2%) Class A, and eight (10.1%) Class C (Table 2).

**Table 2:** Liver function profile in the study cohort ALT: alanine aminotransferase; ALP: alkaline phosphatase; INR: international normalised ratio

Parameter	Number (%)
Total Bilirubin (mg/dL)	
< 2	25 (31.6%)
2 - 4	47 (59.5%)
> 4	7 (8.9%)
Serum ALT (IU/L)	
41 - 100	33 (41.8%)
100 - 200	41 (51.9%)
> 200	5 (6.3%)
Serum ALP (IU/L)	
< 100	23 (29.1%)
100 - 200	48 (60.8%)
> 200	8 (10.1%)
Portal Vein Diameter (mm)	
< 13	15 (19.0%)
13 - 15	58 (73.4%)
> 15	6 (7.6%)
Serum Albumin (g/dL)	
< 3	66 (83.5%)
3 – 3.5	11 (13.9%)
> 3.5	2 (2.5%)
Serum Globulin (g/dL)	
< 2.5	5 (6.3%)
2.5 – 4	63 (79.7%)
> 4	11 (13.9%)
INR	
< 1	67 (84.8%)
1 - 2	10 (12.7%)
> 2	2 (2.5%)
Child-Pugh Score	
Child-Pugh A	12 (15.2%)
Child-Pugh B	59 (74.7%)
Child-Pugh C	8 (10.1%)

Renal assessment and histopathology

The majority of patients exhibited normal renal function at baseline. Serum urea levels were within the normal range in 64 (81.0%) of cases, and serum creatinine was less than 1 mg/dL in 71 (89.9%).

Renal histopathological analysis was performed in 40 patients. Non-glomerular lesions, predominantly involving arterial and tubulointerstitial compartments, were identified in 35 (87.5%) of biopsies. Among glomerular pathologies, IgA nephropathy was most prevalent, observed in 18 (45.0%) of biopsied cases (Table 3).

**Table 3:** Renal function profile in the sample cohort *Individual biopsies could demonstrate more than one glomerular and/or non-glomerular lesion; therefore, lesion counts exceed the total number of biopsied patients.

Parameter	Number of patients (%)
Serum Urea (mg/dL) (n = 79)	
15 - 45	64 (81.0%)
> 45	15 (19.0%)
Serum Creatinine (mg/dL) (n = 79)	
< 1	71 (89.9%)
1 - 2	8 (10.1%)
Renal Biopsy Findings* (n = 40)	
Glomerular Pattern	33 (82.5%)
1. IgA Nephropathy	18 (45%)
2. Membranoproliferative	4 (10%)
3. Diabetic nephropathy	9 (22.5%)
4. Others	3 (7.5%)
Non-Glomerular Lesions	35 (87.5%)
1. Renal vascular injury	30 (75%)
2. Tubulointerstitial injury (acute/chronic)	29 (72.5%)

Glomerular filtration rate analysis

GFRs were calculated and compared using three distinct methodologies (Table 4). The Cockcroft-Gault formula produced the highest average GFR at 97.1 mL/min/1.73m², classifying only three patients with a GFR below 60 mL/min/1.73m². Timed creatinine clearance resulted in an average GFR of 83.6 mL/min/1.73m² and identified five patients with reduced GFR. The CKD-EPI 2021 equation, employed as the primary diagnostic method in this study, yielded the lowest average GFR at 79.1 mL/min/1.73m² and identified the largest number of patients with reduced GFR.

**Table 4:** Categorization of patients into glomerular filtration rate (GFR) subsets using three distinct calculation formulae

GFR formulae	GFR (mL/min/1.73m^2^) n=79			Average GFR
	>90 (n)	60 – 90 (n)	<60 (n)	
Cockcroft-Gault Formula [10]	67	9	3	97.1
Timed Creatinine Clearance-based GFR	48	26	5	83.6
CKD-EPI Creatinine (2021) [8]	39	31	9	79.1

When stratified by hepatic etiology using CKD-EPI 2021 criteria, reduced GFR (<60 mL/min/1.73m²) was identified in nine of 79 patients, corresponding to a prevalence of 11.4% (95% CI: 4.4%-18.4%). The prevalence of reduced GFR differed substantially by etiology (Table 5). Among patients with MASLD, four of 14 individuals (28.6%; 95% CI: 4.9%-52.3%) demonstrated reduced GFR, compared with two of 39 patients with ALD (5.1%; 95% CI: 0%-12.0%).

**Table 5:** Distribution of hepatic etiologies across glomerular filtration rate (GFR) subsets (>90, 60-90, and <60 mL/min/1.73m²) Statistical significance is defined as p < 0.05. Significant p-values are denoted with an asterisk (*). MASLD: metabolic dysfunction-associated steatotic liver disease.

	Number of patients (n) in each group- GFR (mL/min/1.73m^2^) using CKD-EPI Creatinine (2021) [8]			
Etiology (n)	>90 (n)	60−90 (n)	<60 (n)	p-value
Alcohol-Related Liver Disease (39)	21	16	2	Ref value
MASLD (14)	6	4	4	0.039*
Viral Hepatitis (10)	6	3	1	0.441
Autoimmune (4)	1	3	0	1
Wilson’s disease (1)	1	0	0	1
Cryptogenic (4)	2	1	1	0.231
Combined (5)	2	2	1	0.279

Patients with MASLD demonstrated significantly higher odds of renal dysfunction compared to those with ALD (OR 7.4; 95% CI: 1.18-46.4; Fisher’s exact p = 0.039).

## Discussion

The present study found that CLD most commonly affected individuals in their fifth decade, with a mean age of 49.26 years. This finding corresponds with epidemiological studies describing similar age distributions in cirrhosis populations [11]. The observed male predominance is also consistent with the known higher prevalence of CLD among men.

ALD emerged as the leading etiology, followed by MASLD and viral hepatitis. This pattern closely mirrors large Indian and international cohort studies, reinforcing the continued role of alcohol as a major driver of cirrhosis in middle-aged males [12,13].

The biochemical profile - moderate hyperbilirubinemia, modest transaminase elevation, and near-normal alkaline phosphatase (ALP) - corresponds with laboratory patterns described in compensated and moderately decompensated CLD [14]. Profound hypoalbuminemia in more than 80% of patients reflects reduced hepatic synthetic capacity.

Most patients were classified as Child-Pugh Class B, indicating moderate hepatic dysfunction at diagnosis. Many hospital-based cohorts reveal higher proportions of Class C disease due to late presentation [13]. The relatively lower proportion of advanced disease in this study may reflect earlier detection through routine health-check programs.

A central finding of this study is that renal involvement may be present at a subclinical histological stage. The majority of patients had portal vein diameters ≥13 mm, a recognized marker of portal hypertension. Portal hypertension induces systemic circulatory changes that reduce effective arterial blood volume and compromise renal perfusion [15,16].

Despite largely normal serum creatinine and urea levels, the renal biopsy showed significant structural pathology. Non-glomerular lesions, particularly vascular and tubulointerstitial injury, were most common. Similar findings have been reported by Trawalé et al., demonstrating that these injuries precede overt renal dysfunction in cirrhosis [17].

IgA nephropathy was the most frequent glomerular lesion. This association is well established and attributed to impaired hepatic clearance of IgA immune complexes [18,19].

GFR estimation showed that the CKD-EPI equation yielded lower, more conservative values than the Cockcroft-Gault equation. Creatinine-based formulas often overestimate renal function in cirrhosis due to sarcopenia and reduced creatinine production. Mindikoglu et al. similarly showed CKD-EPI provides more reliable estimates in cirrhotic patients [20].

MASLD was associated with a significantly higher prevalence of reduced GFR. This supports growing evidence linking metabolic liver disease with CKD through shared mechanisms such as insulin resistance, inflammation, and microvascular injury [21,22]. The calculated odds ratio further supports this association, demonstrating that MASLD patients had more than sevenfold higher odds of reduced GFR compared with those with ALD. However, the study may be underpowered to detect differences in less common etiologies.

Although severe histological abnormalities were frequent, overt renal dysfunction occurred in only nine (11.4%) patients. This is lower than in cohorts with decompensated cirrhosis, where AKI and hepatorenal syndrome are common [23]. The lower prevalence in this cross-sectional cohort likely reflects earlier disease stages.

Overall, these findings indicate that renal histopathological changes precede detectable alterations in standard blood tests, underscoring the importance of concurrent assessment of hepatic and renal function.

Limitations of the study

There are several limitations to this study. First, because it was done at a single center and used a cross-sectional design, it cannot show cause and effect or track kidney function over time. The sample size of 79 provides useful early insights but remains small and may not reflect the full regional disease burden. Furthermore, the small sample size and the low number of total clinical outcomes (n=9 patients with a reduced GFR) precluded the use of multivariable regression analysis. Consequently, we were unable to statistically adjust for overlapping metabolic confounders such as diabetes mellitus, hypertension, and obesity, which are intrinsically linked to MASLD and act as independent risk factors for renal decline. Future large-scale studies are required to isolate the independent effect of MASLD from these concurrent metabolic variables.

Second, although 40 patients underwent renal biopsy, stringent clinical and coagulation criteria may have introduced selection bias. Individuals with significant bleeding diatheses or thrombocytopenia were less likely to undergo biopsy and may be underrepresented in the histopathological analysis. Additionally, while broad histopathological categories were identified, detailed grading of lesion severity and chronicity indices were intentionally omitted to maintain focus on the primary clinical and etiological objectives of this study. Future dedicated clinicopathological analyses are warranted to deeply characterize these specific chronicity parameters

Finally, renal assessment primarily relied on standard blood tests and measured GFR. Novel early renal biomarkers, such as serum cystatin-C or urinary neutrophil gelatinase-associated lipocalin (NGAL), were not utilized and may have enhanced early detection of kidney injury. Despite these limitations, the study offers valuable baseline data on subclinical renal disease across CLD etiologies and establishes a foundation for future research.

## Conclusions

In this group, CLD mostly affected middle-aged men, with ALD being the most common cause, followed by MASLD and viral hepatitis. While standard blood tests suggested normal kidney function, biopsies showed that many patients had hidden structural kidney damage. IgA nephropathy was the most common glomerular problem, and most non-glomerular findings were tubulointerstitial and vascular injuries.

The CKD-EPI formula gave lower GFR estimates than the Cockcroft-Gault method, suggesting that older methods may overestimate kidney function. MASLD patients had a much higher rate of reduced GFR, showing the need for early kidney screening in this group.

These results highlight the clinical prudence of assessing liver and kidney function in all newly diagnosed CLD patients, even if routine blood tests appear normal. Because significant structural damage was observed in the clinically indicated biopsy subset, early and concurrent screening is paramount to identifying at-risk patients and preventing downstream complications such as hepatorenal syndrome.
